# Quantification of Skin Adaptation to Lower-Limb Prosthesis Use Through Optical Coherence Tomography Angiography and Thermal Imaging: A Review

**DOI:** 10.3390/bioengineering13080877

**Published:** 2026-07-30

**Authors:** Caleb Carle, Molly Baumann, David A. Jack

**Affiliations:** 1 SICEM (Scientific Innovations in Complex Engineering Materials) Research Lab, Department of Mechanical Engineering, Baylor University, Waco, TX 76798-7356, USA; caleb_carle1@baylor.edu; 2 Extremity Trauma and Amputation Center of Excellence, Defense Health Agency, Falls Church, VA 22042-5101, USA; molly.baumann.civ@health.mil; 3 Center for the Intrepid, Department of Rehabilitation Medicine, Brooke Army Medical Center, JBSA Ft. Sam Houston, San Antonio, TX 78234-4504, USA; 4 Oak Ridge Institute for Science and Education, Oak Ridge, TN 37831-0117, USA

**Keywords:** optical coherence tomography angiography, thermal imaging, prosthetics, skin breakdown

## Abstract

Skin breakdown occurs commonly within patients with lower-limb prostheses where the current clinical practice to diagnose skin breakdown is visual inspection by a clinician. Thermal imaging and optical coherence tomography angiography (OCT-A) have been proposed as early detection methods for skin breakdown and hold great promise. OCT-A is a noninvasive imaging modality that outputs a near-infrared optical signal and captures the reflective depth signal. The recorded transient behavior of the vessel area density (VAD), after repetitive dermal loading conditions, has been correlated to skin breakdown. Thermal imaging tracks the temperature profile of the skin surface after repetitive loading conditions and utilizes the temperature time-to-peak (TTP) as a metric of dermal health like VAD. Both methods are sensitive to imaging artifacts, and current resolutions are presented in this work, along with a discussion of the need to standardize data collection and advance image processing methods. Current approaches typically rely on thresholding that produces varying results based on user-selected limits followed by smoothing without any basis of the underlying physics. This review paper presents a need for a constitutive expression that captures the expected physics, thereby strengthening confidence in the use of either thermography or OCT-A, and enabling true clinical applications to aid patient health.

## 1. Introduction

Skin breakdown is a common issue among patients with lower-limb prostheses where the current clinical practice to diagnose skin breakdown is a visual inspection by a clinician. Approximately 63% of individuals with a lower-limb prosthesis experience skin breakdown at least once in their lifetime [1]. When a skin injury goes undetected for too long during prosthesis use, revision surgery may be needed [2]. Often, when visual inspection confirms skin breakdown, symptoms have already occurred, resulting in the clinician recommending limiting prosthesis use. Thus, the quality of life for the patient significantly decreases [3]. This present review manuscript focuses on two noninvasive surface measurement methods for the early detection and quantification of skin breakdown—specifically, thermal imaging, detailed in Section 2.2, and optical coherence tomography angiography (OCT-A), detailed in Section 2.3. OCT-A has found widespread use in retinal imaging [4], and infrared thermography is used in identifying skin disorders and disease monitoring [5]; there have been few studies combining or comparing these approaches for their use in clinical monitoring of skin adaptation to a prosthesis. Prior reviews are limited in that they only evaluate one imaging modality, do not include focus on prosthesis users, or do not fully address the limitations and future directions. This review paper will address each of these components. Both OCT-A and thermal imaging can monitor the response to skin reactive hyperemia, where thermal imaging utilizes infrared emission to assess the transient skin surface temperature profile and OCT-A images the sub-epidermal network to characterize the transient vascular arial density (VAD). Reactive hyperemia is the rapid perfusion of blood through the collapsed vessels from pressure loading upon pressure release.

Thermal imaging utilizes a time-to-peak response of the temperature to monitor skin reactive hyperemia to dermal stresses [6]. However, as described in Section 2.2.1, the skin surface temperature reading can become negatively affected by artifacts caused by environmental temperature fluctuations, patient motion, and skin moisture. Non-uniformity correction methods, presented in Section 2.2.2, have been introduced to aid in reliable skin temperature profiles [7].

To assess the dermal health using OCT-A, a time-to-peak response of the VAD can be used to assess skin reactive hyperemia [8,9]. However, as detailed in Section 2.1.2 and Section 2.3.1, the calculation itself can become negatively altered by artifacts. Artifacts can be caused by patient movement, optical phenomena, incorrect test setups, or alignment issues [10,11]. Correction methods, as detailed in Section 2.3.3, are performed within a software environment after data collection and include approaches such as affine transformation, Frangi vesselness filters, and contrast-limited adaptive histogram equalization to mitigate image artifacts [11,12,13,14,15]. Additional correction methods discussed in the present manuscript include artificial-intelligence-based algorithms within large data sets, optical clearing agents and statistical characterization models [16,17,18,19].

For thermal imaging and OCT-A, there is considerable noise in the processed results for, respectively, the time-to-peak (TTP) temperature and the VAD, and thus curve fitting and smoothing methods are often used to identify critical points in the resultant curves.

Based on the work presented in the current manuscript, it is clear that thermal surface imaging and OCT-A have significant potential to identify trends between skin reactive hyperemia and either the skin surface temperature profile or the VAD. It is also evident that improvements are needed in the analysis of the captured data as there remains significant variability between clinical setups, operator preferences, analysis approaches, and user-selected thresholding parameters. The findings of this manuscript show the need for further studies to correlate the captured data to physics-based models for proper interpretation.

### 1.1. Postoperative Care and Skin-Prosthesis Adaptation

Given the serious consequences of skin breakdown, meticulous postoperative care and the promotion of healthy skin-prosthesis adaptation are essential to preventing these complications and ensuring successful long-term outcomes. The postoperative sequence, provided by Krajbich et al., is often employed [20]. The first step of this postoperative sequence states that directly after surgery, a soft dressing is applied to the postoperative site to cover the suture line and hold the incision dressing in place. After a few days, a vacuum-assisted closure dressing is placed for 3–5 days to promote cell division, growth factor production, and angiogenesis. Krajbich et al. continue by recommending that, following the use of this dressing, a hydrofiber dressing consisting of soft nonwoven sodium carboxymethylcellulose fibers impregnated with ionic silver is applied for up to 7 days. This helps to retain moisture while providing antimicrobial benefits from the silver. Controlling postoperative edema is imperative for the health of the limb, which is often satisfied through compressive dressings, including polymer gel socks and silicone liners. Shortly after the compressive dressing is applied, a protective (rigid) dressing is placed by a prosthetist with padding over the bony prominences. Krajbich et al. state that this promotes quicker rehabilitation time, reduces edema, and decreases postoperative complications. The rigid dressing is used for 2–3 weeks to prepare the limb for an immediate postoperative prosthesis (IPOP). After the IPOP fitment, the patient’s rehabilitation process begins where partial weight bearing gradually increases based on the patient’s strength, cooperation, and pain levels. During this stage, the skin breakdown risk increases and is closely monitored. Depending on the patient’s limb status and care needed, it takes 2–4 weeks after wound healing to begin prosthetic fitment and therapy. After approximately 3 months, a custom-fit prosthesis is provided to the patient. Throughout the use of the long-term prosthesis, the patient can experience prosthesis-related skin breakdown from poor socket fitment. Krajbich et al. note that due to the extensive time, effort, and costs involved with the postoperative care, it is imperative that the prosthesis can be utilized by the patient for the longest term possible. This will benefit the patient by enabling mobility which can improve quality of life and reduce the time and resources required to make a new prosthesis [3].

### 1.2. Skin Breakdown Mechanisms

**Figure 1:**
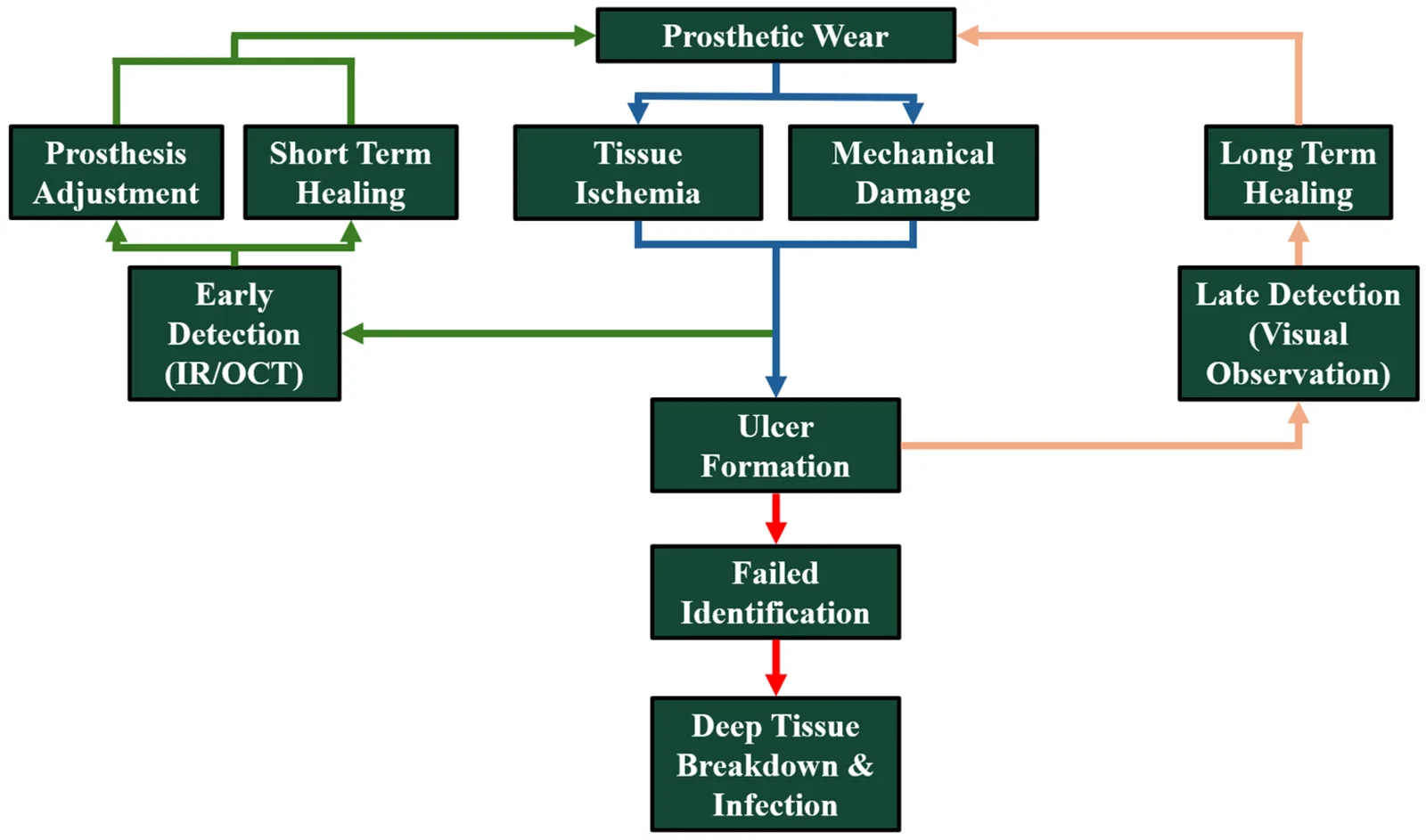
Overview of skin breakdown and corrective actions from lower-limb-prosthesis usage. (blue path) standard prothesis use resulting in tissue damage, (red path) when no corrective action is performed, (orange path) current clinical options, and (green path) alternative corrective path enabled by OCT and IR.

Individuals with lower-limb prostheses commonly experience skin breakdown as a result of wearing their prosthetic device [9,21,22,23]. Traditional socket wear which leads to skin breakdown is depicted in Figure 1 from the blue path to the orange path. During the swing phase of the individual’s gait cycle, the prosthesis tends to pull towards the ground because of the centripetal force [24]. When the prosthesis contacts the ground during the stance phase of the gait cycle, the prosthesis pushes toward the body of the individual. This repetitive motion creates a pistoning effect, creating high shear stress on the residuum which can lead to skin breakdown [25]. Prolonged mechanical loading at the prosthetic socket–skin interface can lead to progressive skin tissue damage, ultimately resulting in skin breakdown. Clinical observations indicate that breakdown commonly initiates at regions experiencing combined pressure and shear, particularly over bony prominences. This phenomenon is typically reflected as a skin ulceration [26]. If the breakdown is not caught sufficiently early, the red path shown in Figure 1 ensues, causing prolonged and potentially life-altering damage. Poor socket fit can exasperate this mechanical environment and accelerate skin breakdown especially over bony prominences and other high-pressure sites. The non-volar skin of the residual limb is not structurally adapted to tolerate such sustained mechanical loads [27].

Typical symptoms identified by clinicians involve the visual observation of skin breakdown such as blisters, lesions, and irritation. Skin breakdown can progress further due to direct cellular deformation and tissue ischemia [28]. Tissue ischemia occurs when the blood flow to the tissue is interrupted due to applied pressure to the skin. This alters the cellular metabolism causing the local pH level to decrease, damaging the surrounding cell’s membranes [28]. This effect can lead to rapid cell necrosis, which if not given time to heal, can spread throughout the tissue and lead to skin breakdown [28]. Prosthesis use can result in both mechanical damage and/or tissue ischemia, shown by the blue path in Figure 1. The tissue ischemia and/or mechanical damage then leads to skin ulcer formation, which is a primary driver of deep tissue breakdown and infection. Skin breakdown falls into three etiologies: friction injury, maceration injury, and pressure injury [29]. Combining these three etiologies together increases the risk of skin breakdown. Friction injury is caused by the shear stresses endured by the skin. Maceration injury is caused by increased moisture from sweat, which softens the skin and reduces its resistance to mechanical stress. Lastly, pressure injury is the result of normal stresses on the skin. When skin lesions, irritation, and ulcers are detected by the patient or visual observation by a clinician, it is often recommended that the patient pauses use of their prosthesis until the skin is fully healed, as shown in the orange path of Figure 1. This is not the desired outcome, and this prevents the prosthesis user from doing their normal daily activities for extended periods of time. The prosthesis user then gradually returns to their normal daily activities using their prosthesis. This impact on the reduced mobility can quickly degrade the quality of life of the individual [3], reducing the benefits of the prosthesis to a large extent. The present manuscript seeks to highlight an alternative path to identify the onset of tissue damage prior to ulcer formation, indicated by the green colored path in Figure 1, using thermal imaging or OCT-A. The expectation is that this early detection will occur before the user is forced to stop the use of the prosthesis and enable the clinician to take less severe corrective action, preventing the need to stop prosthesis use and thereby enhance the quality of life of the prosthesis user.

### 1.3. Skin Breakdown Assessment and Quantification Methods

**Figure 2:**
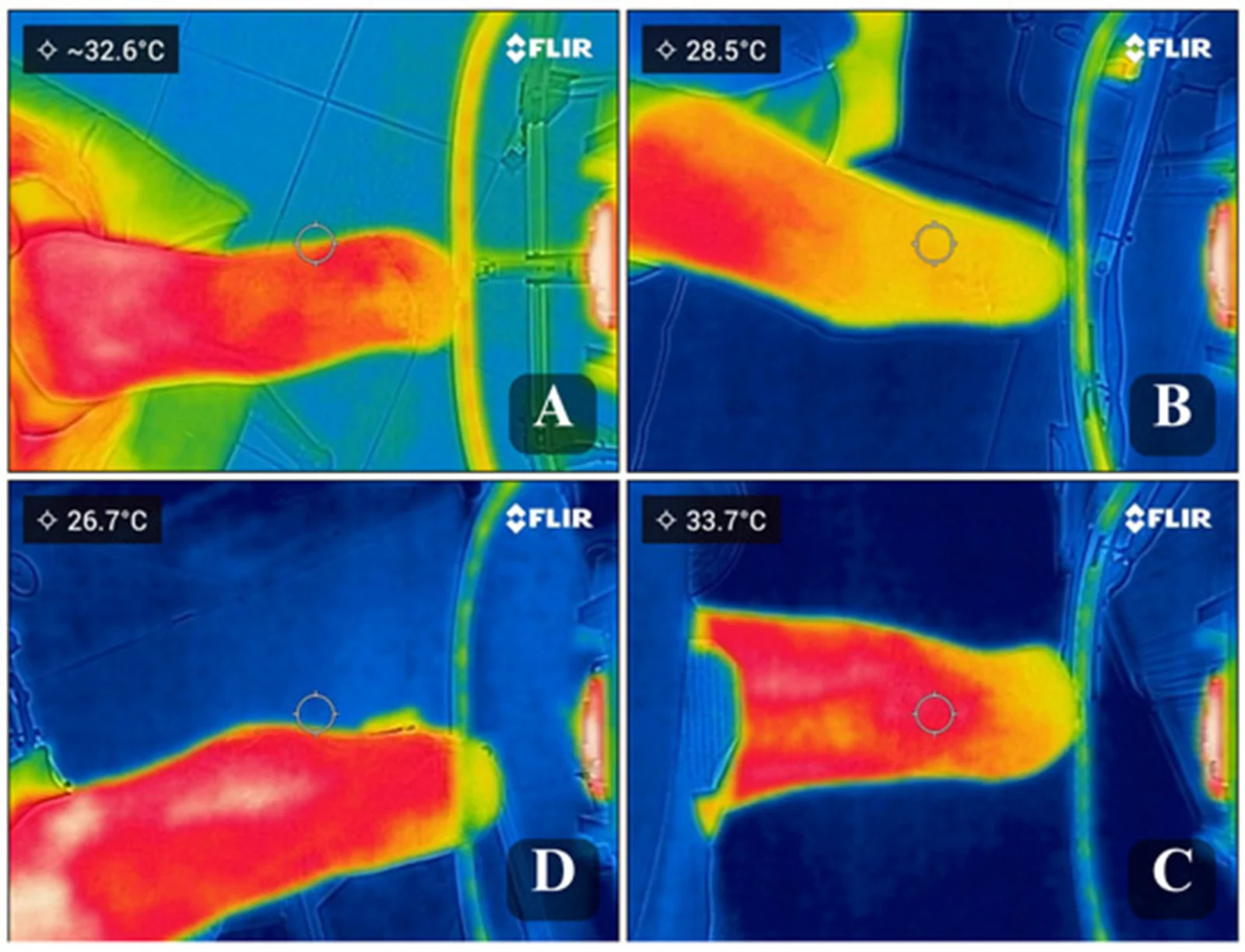
Thermal image of a patient’s residual limb after being subjected to a thermal blanket. (**A**) Anterior view, (**B**) lateral view, (**C**) posterior and (**D**) medial view. Reproduced from [33] and distributed under the Creative Commons Attribution (CC BY) license.

Currently, the detection and diagnosis of skin issues are limited as these are most often identified by the visual observations of a trained clinician, making it too late to take more preventative measures. Early detection methods have been proposed to prevent skin breakdown events before symptoms become prominent, but studies are still lacking in this area. The use of thermal imaging to detect skin surface temperature after repeated loading has demonstrated efficacy [6], while others have used thermal imaging to assess baseline temperature and temperature during activity [30,31,32]. Pizarro et al. utilized thermography to monitor the skin’s thermoregulation for a patient that wore a lower-limb prosthesis by subjecting the limb to a thermal blanket and then observing the skin’s response after the heat was removed, as shown in Figure 2 [33]. Although Pizarro et al. do not subject the participant to any mechanical loading, they state that skin health can be reflected by swift thermoregulation, which indicates well-functioning blood vessels.

To gather a response to mechanical loading from the prosthetic socket structure, thermal imaging typically follows a loading cycle on the residual limb’s skin with the temperature of the area of interest being collected at different times post-loading. The skin’s hyperemic response indicates its ability to restore blood following mechanical loading and reflects skin tissue integrity. Delayed hyperemia signals damaged skin tissue and its increased susceptibility to breakdown.

**Figure 3:**
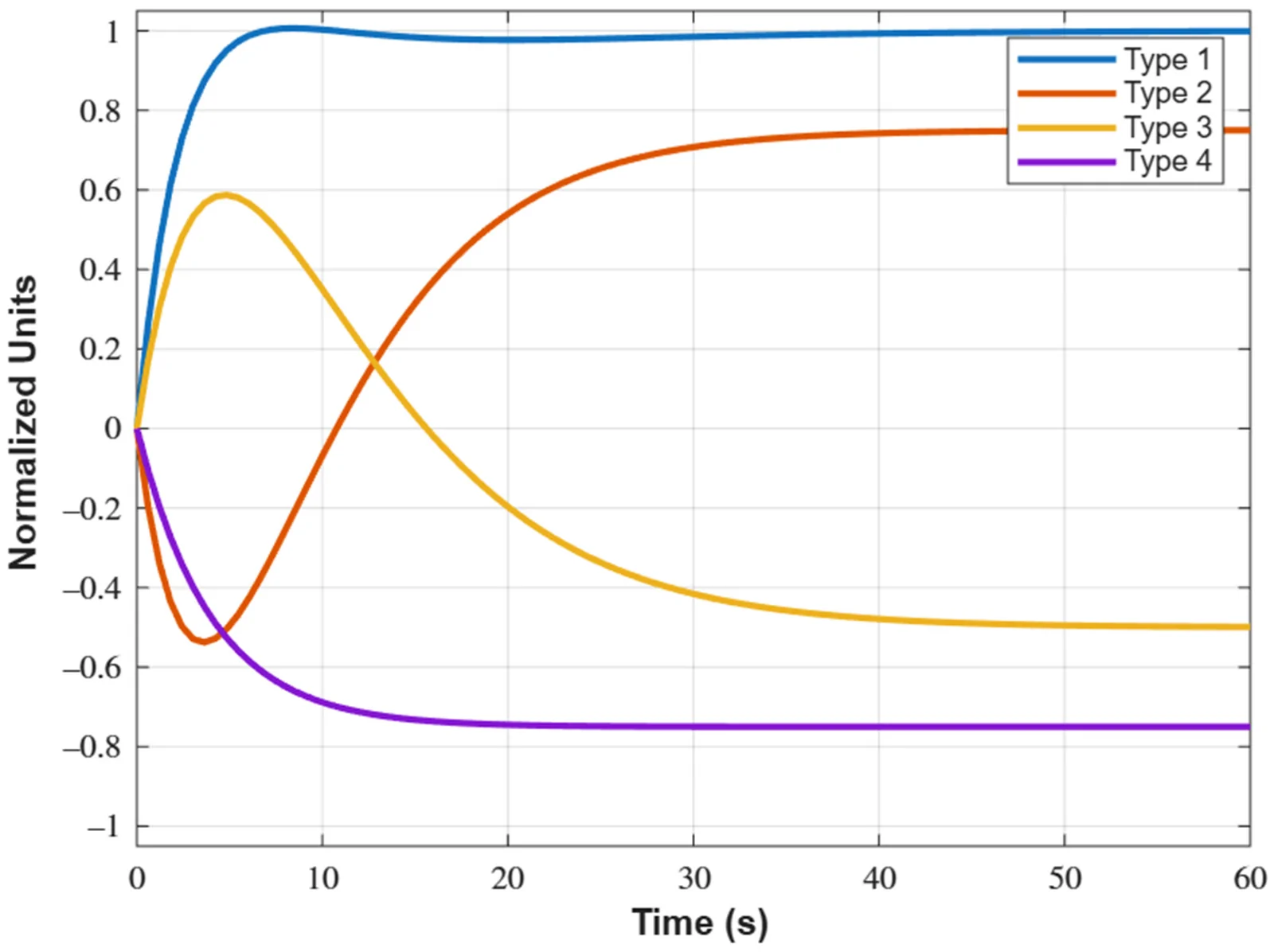
Four types of typical thermal profiles of a patient’s residual.

Thermal imaging cameras focus infrared radiation onto a pixel array detector called a bolometer which converts a thermal signal into an electrical signal [34]. The temperature profile over time can provide insight into the skin response to stress, where the regional maximum temperature is typically recorded for analysis. The peak of the regional maximum transient temperature is termed the time-to-peak temperature response (TTP). According to Sanders et al., TTP has been suggested to quantify skin adaptation where a slower TTP indicates poorly adapted skin and a faster TTP denotes adapted skin [6]. Sanders et al. reason that blood perfusion achieves a faster TTP in adapted skin because of prolonged exposure to pressure induced angiogenesis, causing the vascular plexus to enlarge. Due to an enlarging vascular plexus, the cross-sectional areas of the blood vessels increase, allowing for a greater blood transport rate. IR cameras are helpful in capturing skin health because of human physiological principles that generate heat. Since metabolic processes—such as muscle activation—generate heat as a byproduct, the heat is transferred to nearby blood vessels through conduction. Blood heat transfer also experiences convection when carrying blood to different locations of the body [35]. When a larger blood flow rate is experienced at a location, such as the dermis of the skin due to the increased vessel diameter, the skin experiences a large rate of heat transfer through conduction. The TTP has been used to assess this thermal profile from the hyperemia response [6], where they observed four different types of standard responses for the TTP, such as shown in Figure 3. In general, a high skin surface temperature indicates a hyperemia response as seen in Type 1 and 3, and a low skin surface temperature as seen in Type 2 and Type 4 demonstrates tissue ischemia as occluded blood flow limits temperature conduction. For curve Type 1, there is an increase to a maximum temperature and then a small decrease to stabilization where the final temperature is higher than the initial temperature. Occasionally, there will be stabilization without a decrease in temperature. For Type 2, an initial decrease in temperature is noted, followed by a rapid increase in temperature to stabilization. For Type 3, an initial increase in temperature occurs followed by an exponential decrease in temperature to stabilization. This curve type is an expected example of healthy tissue. For curve type 4, there is a rapid decrease in temperature until stabilization is well below the starting temperature.

Another early-detection method for skin adaptation would be through the usage of optical coherence tomography angiography (OCT-A), a subset of optical coherence tomography (OCT). OCT is commonly used within ophthalmology to image microvessels in the eye [36,37] but has more recently been deployed in imaging skin [8,9,38,39,40]. OCT methods utilize infrared light signals and measure the interference between a source light and a reflected light to gauge depth and particle motion. OCT is typically used to generate cross-sectional images of the tissue microstructure, whereas OCT-A, an extension of OCT, detects the moving blood cells from within a series of OCT scans [41]. Capillary blood flow and structural features such as skin surface roughness recorded from, respectively, OCT-A and OCT, can classify skin injuries and pathological conditions such as psoriasis, burn injuries, or prosthetic-induced breakdown [42,43]. OCT can also characterize collagen content in skin, which can identify fibrosis and monitor treatments [44]. The measurement of the microvascular network within the dermal layer is believed to indicate characteristics of the skin that clinicians can employ to diagnose patients with prosthesis-induced skin breakdown. OCT-A can capture vascular structures and features 1–2 mm deep into the skin with as fine as a 1.7-micron resolution [45]. One metric extracted from the OCT-A measurement regarding tissue health is the VAD, which is found in the dermal layer of the skin, approximately 1–2 mm deep into the skin. Since blood flow reduces by an average of 63% in damaged skin compared to healthy skin, changes to the VAD parameter can be correlated to tissue health directly [46]. OCT-A has been able to achieve a highly repeatable extraction of VAD, showing that it is promising for this application [47].

To better understand how VAD and TTP are obtained, it is important to consider the typical experimental set-up and imaging methods used in OCT-A and IR studies of residual limb tissue. The patient is seated on a physical therapy table with the residual limb supported to limit movement. Prior to imaging, the patient’s skin is subjected to a period of controlled mechanical loading at clinical regions of interest where skin breakdown may often be seen. The OCT camera or the IR camera is then placed proximally to the location of interest to begin the alignment procedure. After alignment, the loading cycle begins on the patient’s skin. The patient is typically positioned on a physical therapy table with the residual limb supported while the camera is oriented orthogonally to the skin surface in close proximity [48].

**Figure 4:**
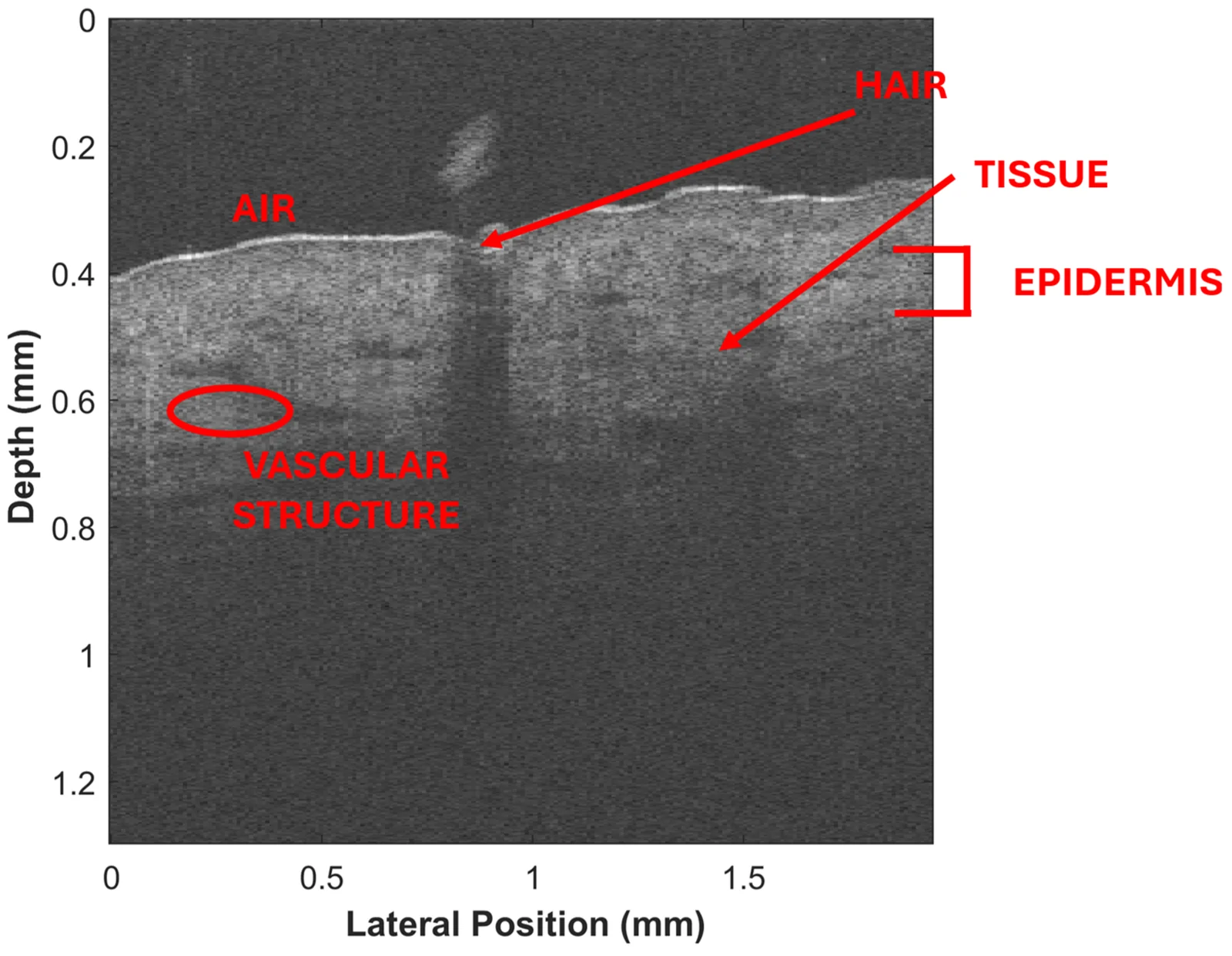
Representative B-scan from a single OCT scan of a patient’s limb.

For IR imaging, researchers often manually adjust the infrared camera position and focus relative to the residual limb using the live imaging view. Conversely, for OCT imaging, a B-scan is typically used to facilitate OCT lens positioning, alignment, and quantitative assessment of the skin tissue at the residual limb such as that shown in Figure 4 taken by the present authors. This figure shows a single OCT B-scan of a patient’s limb. These B-scans provide a cross-sectional view of the dermal structure and can serve as the modality for quantifying epidermal thickness [27]. Notice in the figure that individual features can be observed, such as the epidermis, individual hair follicles, and even some substructure of the vascular network.

**Figure 5:**
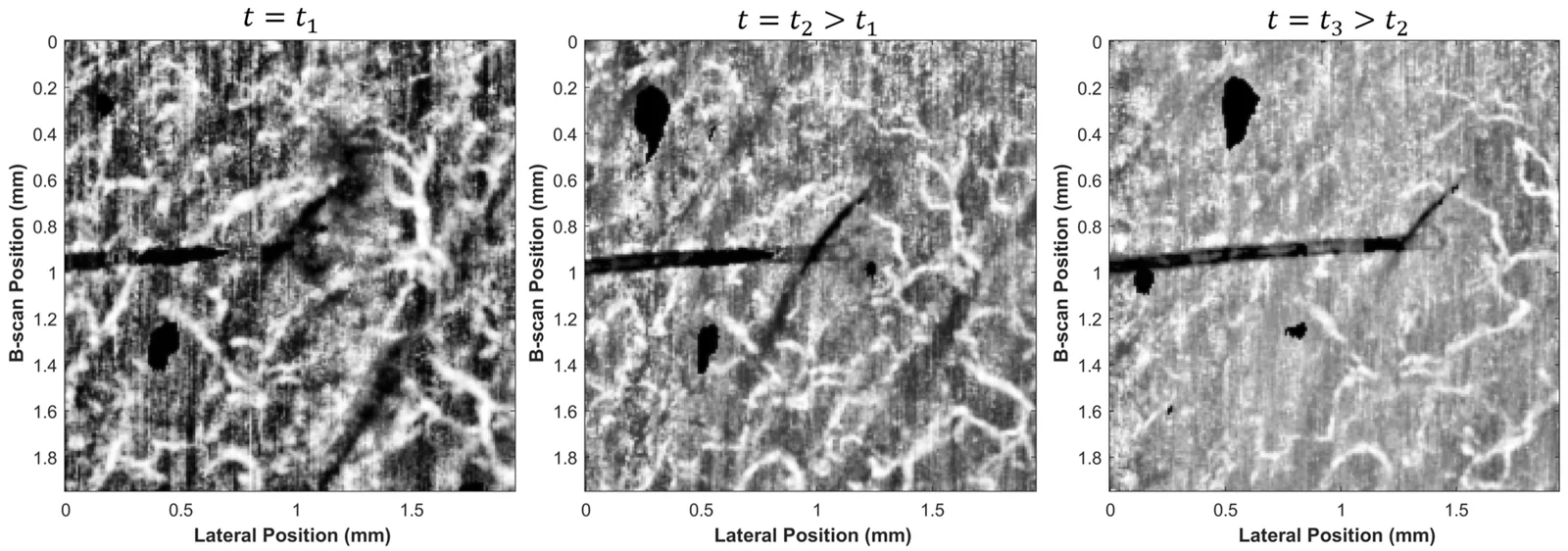
Representative OCT-A C-scan of a patient’s limb after contrast adjustment.

Real-time B-scans enable adjustment of the OCT camera position to ensure that the optical wavelengths are oriented orthogonally to the skin surface and positioned in close proximity to the location of interest. The high-contrast visualization of the air and tissue interface provides a reliable reference for confirming satisfactory focus and alignment prior to data acquisition. Once aligned properly, B-scan images can be used to identify the epidermal layer based on its contrast relative to underlying tissue [38]. Epidermal thickness can be quantified by measuring the axial distance between the air–skin boundary and the epidermal-dermal junction within the B-scan. After acquiring a B-scan and confirming that the B-scan is satisfactory, Swanson et al. [9] performed a C-scan at the region of interest to ensure that the microvessels can be clearly visualized. They note that the B-scan provides a cross-sectional view of the tissue structure, whereas the C-scan captures an en face view of the area, providing a different perspective of the vascular network. For example, a collected series of C-scans can create a composite visualization of tissue structure and the microvascular network, such as that depicted in Figure 5, taken by the present authors. This sequential imaging ensures that the vessels identified in the B-scan are accurately represented in the C-scan and that the image quality is sufficient for data capture. In Figure 5, the vessel size changes with time after loading. During $t_{1}$, the vessels are larger in area due to the high magnitude of blood perfusion. Considering $t_{2}$ and $t_{3}$, the vessels reduce in area due to the decreasing magnitude of blood perfusion.

## 2. Existing Methodologies for Quantifiable Skin Tissue Health Monitoring

Thermal imaging and OCT-A have been proposed as early detection methods for skin breakdown and hold great promise [8,9,40]. In this section the two approaches are explained in detail along with their individual benefits and drawbacks.

### 2.1. Broad Challenges with IR and OCT-A

Both methods have several significant challenges preventing their acceptance in clinical use. Some challenges are unique to each method, such as resolution, environment, thresholding selections and modality. Some challenges are shared between methods, such as a dependence on human factors during setup and the individual patient’s long-term and recent history.

#### 2.1.1. IR Imaging

While valuable and reliable for detecting temperature variations and assessing skin thermoregulation, IR imaging faces several inherent limitations that can compromise its clinical utility. Ambient temperature fluctuations can affect the temperature reading of the imaging surface and a change in relative humidity can alter the temperature profile of the imaging surface [49]. Additionally, IR imaging only provides skin-surface-level information without revealing vascular dynamics or deep tissue conditions that may be contributing to observed temperature changes. This can make it difficult to distinguish between bodily thermal fluctuations and the targeted early pathological changes occurring below the epidermis. Skin temperature is also influenced by factors such as metabolic rate and recent activity, further complicating interpretation from only superficial readings. As a result, while IR can indicate skin thermal patterns like inflammation and hyperemia, it lacks the specificity and subcutaneous imaging capabilities that OCT-A has for early detection of skin breakdown.

#### 2.1.2. OCT-A Imaging

To reliably calculate the VAD within the imaging window, the captured image must be of high quality to do so. It also requires extensive computational and processing time, which is prohibitive in a clinical setting. The technique also requires careful alignment and positioning, which can limit clinical accessibility because of the large space requirements for the positioning equipment. The requirement for alignment between the OCT camera and the skin surface makes it challenging to begin the image capturing process following the loading cycle. Beginning image capturing as soon as possible after the loading cycle is imperative because the reactive hyperemia response begins instantaneously after mechanical pressure is released. During the loading cycle, the patient’s skin surface could potentially move from the previous location during the OCT alignment, which can lead to poor image quality. Lastly, OCT-A can succumb to physiological and optical artifacts during the imaging process due to its sensitivity and high resolution.

#### 2.1.3. Patient Motion Management

To isolate the limb that will be imaged, researchers often combine the use of therapy-foam blocks and a vacuum splint to prevent accidental translational and/or rotational motion of the limb. A pressure sensor can also be used to identify the local pressures of the skin surface to allow for better repeatability across multiple patients, since the applied pressure is standardized [50]. The applied pressure aids in fixing the skin surface in place to prevent the region of interest from changing locations due to tremoring or twitching. Software algorithms that mitigate patient-induced motion artifacts represent another approach that can complement mechanical and electromechanical stabilization systems. Ricco et al. proposed an affine transformation procedure which uses a reference image and a test image to solve for a rotational/translation matrix to use to correct the test images [11].

### 2.2. Thermal Imaging (IR)

Infrared radiation (IR) thermal cameras use specialized lenses to direct infrared energy onto a detector array made from micrometer size pixels containing materials sensitive to IR wavelengths called microbolometers [34]. The materials typically used are vanadium oxide (VOx) and amorphous silicon (a-Si). Microbolometers change their resistance when interacting with incident radiation of the heat source just as thermistors do. The change in resistance values causes a change in voltage as a function of temperature [51]

(1) $$V=I_{b}\frac{dR}{dT}\Delta T$$

where V is the voltage, $I_{b}$ is the biasing current, R is the resistance, and ΔT is the change in temperature at the specific pixel location. The sensitivity and response speed of the microbolometer are dependent on their small size and the large temperature coefficient of resistance, denoted as [51].

(2) $$\alpha =\frac{1}{\rho }\frac{d\rho }{dT}$$

where α is the temperature coefficient of resistance, ρ is the resistivity, and T is the temperature. The heat balance equation for a bolometer is [51].

(3) $$C\frac{d\left(T+\Delta T\right)}{dt}+G\left(T+\Delta T-T_{0}\right)=I_{b}^{2}\left(R+\Delta R\right)+W_{0}sin(\omega_{m}t)$$

where C is the thermal capacity of the bolometer, T is the temperature of the bolometer, ΔT is the change in temperature, $T_{0}$ is the ambient temperature, G is the thermal conductance between the bolometer and surrounding thermal environment, $I_{b}$ is the biasing current, R is the resistance across the bolometer, ΔR is the change in resistance across the bolometer, $W_{0}$ is the radiation power dissipated by the bolometer, and $\omega_{m}$ is the modulation frequency multiplied by 2π. The leftmost term represents heat accumulation, the second term is the heat that flows out of the bolometer, and the right-hand side terms represent the incoming heat flow.

After solving for the change in temperature, one obtains [51].

(4) $$\Delta T=\frac{W_{0}}{G_{e}^{2}+\omega_{m}^{2}C^{2}}sin(\omega_{m}t-\tan^{-1}\frac{\omega_{m}C}{G_{e}})+ce^{\frac{{-G}_{e}t}{C}}$$

where $G_{e}$ is the effective thermal conductance and c is an arbitrary constant. The change in temperature functions occur on each pixel element of the microbolometer array.

Although the temperature calculation for an individual pixel location is based on well-established radiative heat transfer principles, accurately measuring the temperature of human skin introduces several additional sources of uncertainty. Unlike ideal blackbody surfaces, human skin exhibits spatially varying emissivity that can change with sweat and must be considered when interpreting infrared temperature measurements as the subtle change in detected temperature may be diagnostically significant in clinical applications. Fortunately, for clinical use, skin pigmentation, patient height and weight do not affect skin surface temperature measurements [52].

#### 2.2.1. IR Camera Measurement Challenges

Although thermal imaging is a robust measurement technique, it tends to experience low image contrast and low resolution due to the low frequency properties of the infrared spectra [7]. Infrared radiation is fundamentally less sensitive to fine spatial variations compared to light, which limits the ability of thermal cameras to detect small or subtle temperature differences on the skin. A temperature accuracy of ±0.2 °C can have a significant therapeutic impact and many IR cameras do not meet this condition [53]. IR cameras also require extensive processing methods to mitigate non-uniformity noise which affects the accuracy of the temperature measurement, signal-to-noise ratio, resolution, and contrast of the IR camera [7]. They have also been shown to exhibit false temperature fluctuations that do not represent actual physiological changes due to changing ambient temperature conditions and relative humidity impacts on the skin surface [49].

#### 2.2.2. Methods to Mitigate IR Measurement Challenges

During processing, non-uniformity correction is typically required to de-noise the signal produced by the detector array. The non-uniformity correction equation often used is given by Hou et al. [7] as

(5) $$NU=\frac{1}{V_{avg}}\sqrt{\frac{1}{M\times N-(d+h)}\sum_{i=1}^{M}\sum_{j=1}^{N}{\left(V_{ij}-V_{avg}\right)}^{2}}$$

(6) $$V_{avg}=\frac{1}{M\times N-(d+h)}\sum_{i=1}^{M}\sum_{j=1}^{N}V_{ij}$$

where M is the number of rows of the detector array, N is the number of columns of the detector array, $V_{ij}$ is the output voltage of pixels in row i and column j of the detector array, $V_{avg}$ is the invalid detection element of the output voltage of the detector array, d is the number of dead pixels in the array, and h is the number of overheated pixels in the array. One should further note that $V_{avg}$ is the parameter used to compare the non-uniformity of the imaging plane. Additionally, thermal images can undergo calibration-based and scene-based corrections to amend non-uniform temperature profiles. There are several strategies for correcting non-uniformity noise in IR imaging. The methods are typically categorized into calibration-based and scene-based approaches [7]. Calibration-based techniques, including one-point, two-point, multi-point, and interpolation correction rely on pre-determined reference temperatures to adjust detector readings. Conversely, scene-based methods such as time domain high-pass filtering, Kalman filtering, neural networks, and alignment-based approaches use information from the captured scene itself to reduce non-uniformity artifacts [7]. Collectively, these methods enhance temperature measurement accuracy and mitigate errors introduced by detector variability and environmental influences.

#### 2.2.3. TTP Plots from IR Measurement

**Figure 6:**
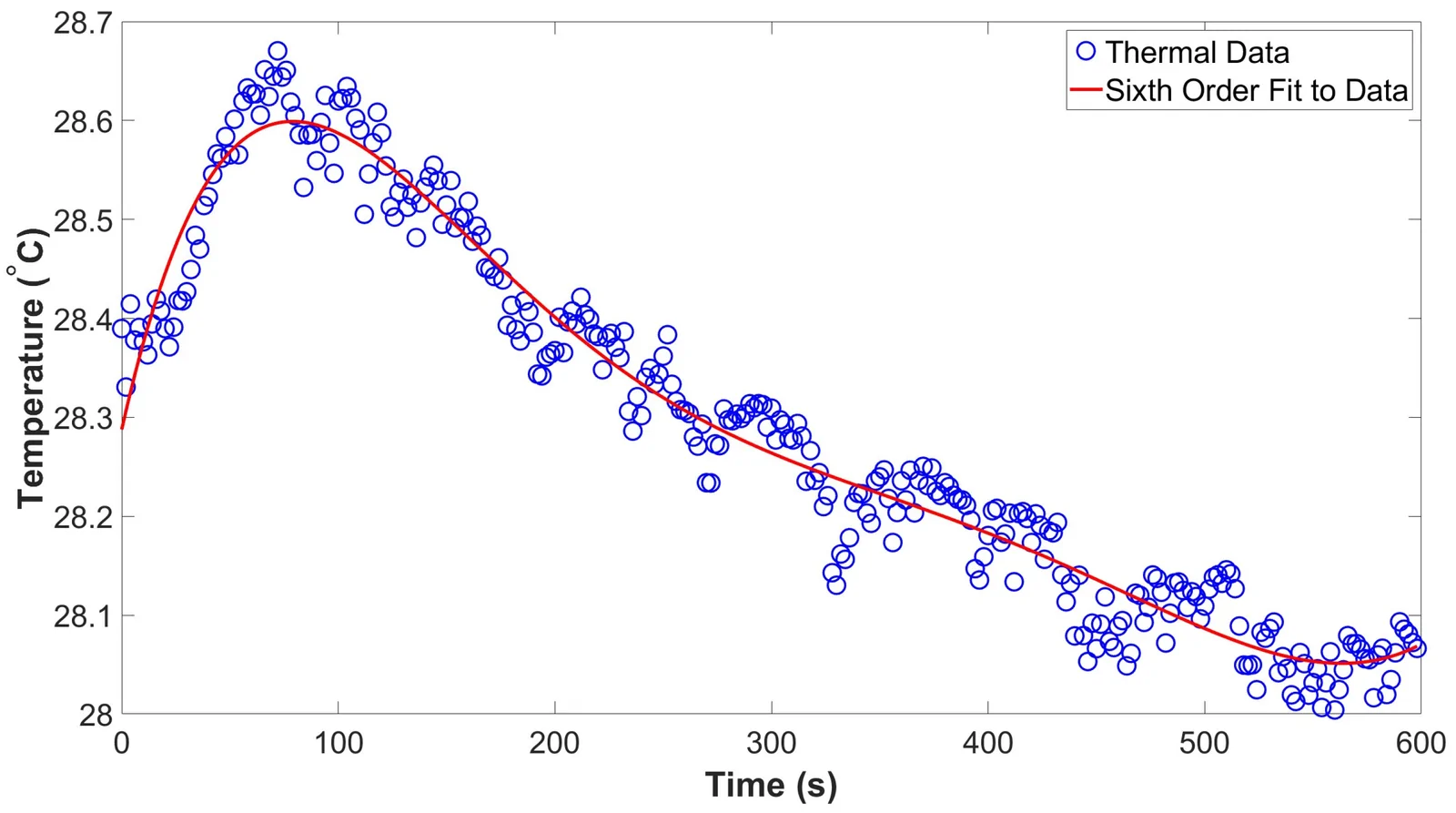
TTP plot of patient’s residual limb after repetitive loading conditions.

IR thermography is used to assess dermal blood perfusion and thermal recovery following mechanical loading by analyzing TTP plots, as shown in Figure 6. After the removal of the external load, the skin undergoes reactive hyperemia which produces a transient increase in surface temperature as blood flow perfuses through the microvascular network. The skin temperature reading is from the conduction of heat from the blood through tissue to the skin surface where infrared radiation is emitted to the IR camera. Time-to-peak is defined as the time interval between the load removal and 70% of the maximum recorded skin temperature, providing a quantitative measure of the rate of vascular recovery [6]. Healthy tissue is believed to exhibit a rapid thermal response with a shorter TTP, whereas prolonged TTP values may indicate impaired microvascular function or early tissue damage [6]. TTP plots are an important tool for evaluating tissue viability, assessing susceptibility to pressure-induced injury, and monitoring the effectiveness of medical interventions or prosthesis adjustment in populations at risk of skin breakdown.

### 2.3. Optical Coherence Tomography (OCT) and Optical Coherence Tomography Angiography (OCT-A)

**Figure 7:**
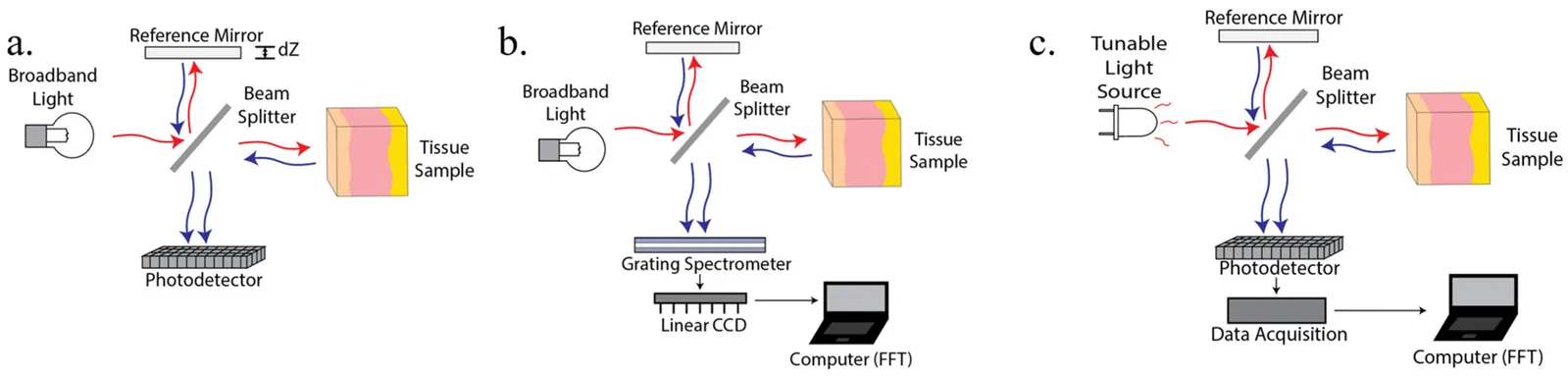
Optical coherence tomography (OCT) types: (**a**) time-domain OCT, (**b**) spectral-domain OCT, and (**c**) swept-source OCT.

OCT is a non-invasive imaging modality that projects a light signal within the near-infrared window (~900–940 nm) into the skin and measures the reflective signal using a detector and computer for processing. Traditional time-domain OCT (TD-OCT), depicted in Figure 7a, utilizes a beam of light that is emitted from a photodiode and enters a beamsplitter that splits the light beam into two beams: one to a reference mirror that is adjusted by a distance $d_{z}$ corresponding to the desired depth of interest within the sample, and another beam passing through the lens to the sample using an interferometer [54]. The two beams of light are then recombined by the beamsplitter, creating an interference pattern. The intensity of the interference signal is measured by a photodetector as a function of optical path delay. Due to the low coherence of the light source, interference is observed when the path length difference matches that of the sample reflector. OCT-A takes a series of OCT images over the same region and uses methods to look at changes within the images indicative of blood flow, such as the method of speckle density, to present images of the differences in intensity fluctuations between static and dynamic objects [55]. The interferometer within OCT performs a raster scan over the imaging window and applies a speckle pattern to detect how the reflection of each speckle changes over the acquisition time window. Spectral-domain OCT (SD-OCT), depicted in Figure 7b, does not require the mechanical movement $d_{z}$ of a reference mirror that TD-OCT does. Rather, it extracts all interference spectra that are encoded in one shot over a range of frequencies using a grating spectrometer and a linear CCD. A computer then performs a Fourier transform to extract the A-scan that represents intensity as a function of depth. SD-OCT greatly improves the signal-to-noise ratio (SNR) and the imaging speed compared to TD-OCT [56]. However, the signal decreases heavily compared to TD-OCT as depth increases [57]. It is also more expensive due to the complex hardware it requires. Swept-source OCT (SS-OCT), depicted in Figure 7c, replaces the spectral domain broadband light source that SD-OCT has with a tunable light source, or a laser, that sweeps at a pre-determined wavelength over a spectrum of wavelengths that is then captured by a photodetector. The photodetector detects the interferometric signal as the tunable light source sweeps over the numerous wavelengths sequentially. One sweep is passed as an A-scan within the data acquisition module where the reflections are detected. A Fourier transform converts the interferometric signal of the A-scan to a reflection depth signal. The A-scan then follows a raster pattern along sequential locations to generate the B-scan. Although SS-OCT is the fastest and most sensitive OCT generation, it tends to be more expensive computationally and monetarily. All three OCT types depicted in Figure 7 share common elements, including a beam splitter that divides light between reference and sample arms, with the recombined beams generating interference patterns that are digitally converted depth-resolved structural information from the sample. When SS-OCT is compared to TD-OCT and SD-OCT, the measurement sensitivity, SNR, and image acquisition time is increased [56]. However, SS-OCT requires more expensive components and achieves a lower axial resolution, thus making SD-OCT the most common [56].

#### 2.3.1. Issues with OCT-A Image Acquisition

The acquisition of high-quality OCT-A images is often hindered by a combination of physiological and optical artifacts that must be carefully managed during the scanning and processing procedure. One of the physiological sources that produces artifacts is patient motion, caused by tremoring or subtle movements of the limb that can cause the region of interest to translate and/or rotate during image acquisition. This motion artifact produces a large pixel intensity variation which creates a blurring effect and an inconsistent contrasting profile. Another physiological source is the patient’s hair that blocks the field of view of the camera which eliminates regions of the skin tissue to contribute to the VAD measurement. OCT image acquisition can also be affected by various optical artifacts, such as those listed in Table 1, which present significant challenges to clinicians analyzing OCT-A images. Each artifact arises from limitations in scan alignment, focusing, light propagation, or Fourier-domain reconstruction, and can lead to reduced image quality, loss of structural detail, or misrepresentation of skin tissue morphology. Understanding, identifying, and correcting these artifacts ensures accurate image interpretation and minimizes errors in quantitative OCT-A analysis.

#### 2.3.2. OCT Structural and Variance Data Types

**Figure 8:**
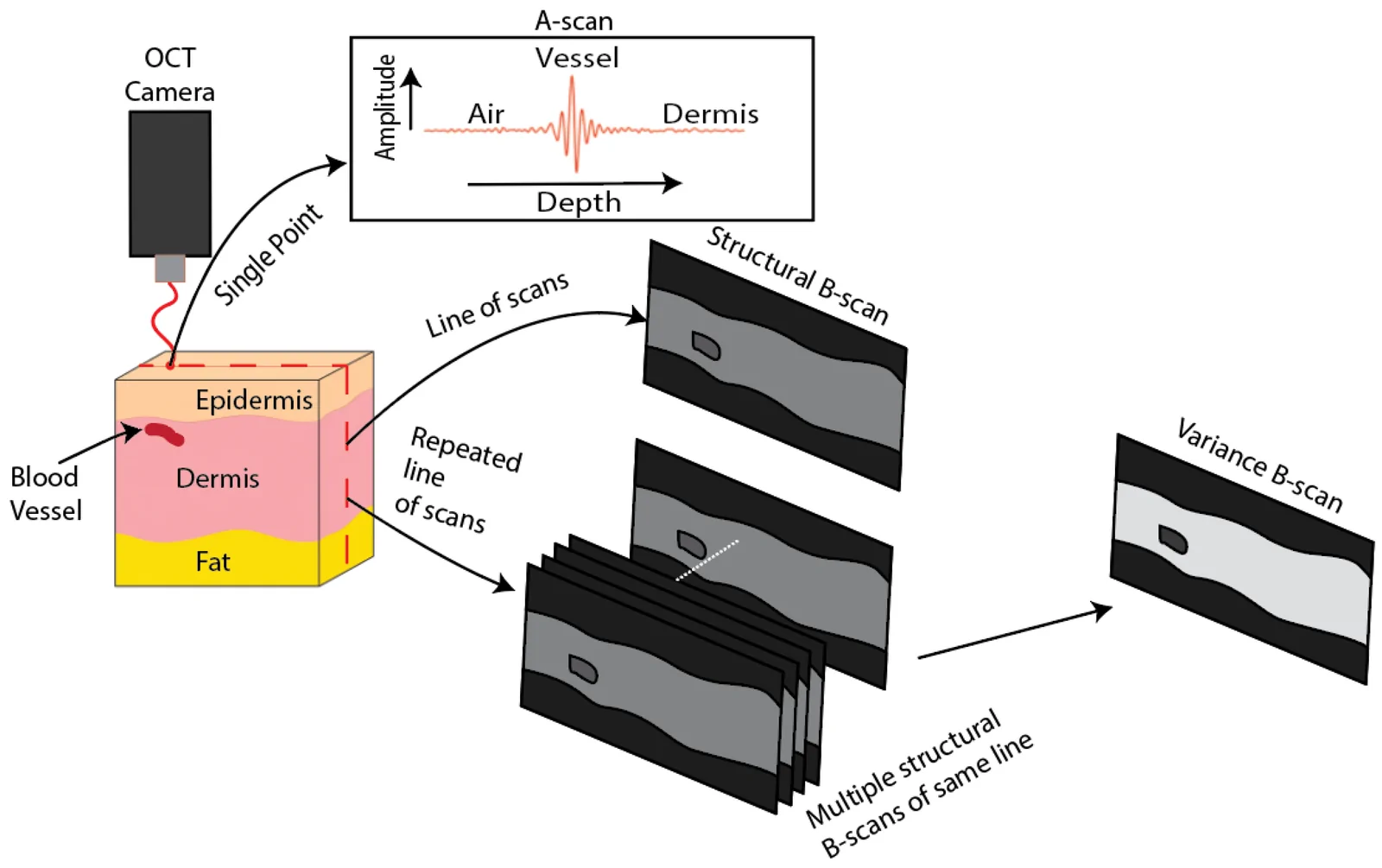
Structural OCT and OCT-A (variance OCT).

OCT has many different data types depending on the acquisition and processing methodology. The two main data types for imaging dermal tissue characteristics and microvascular geometry are structural data and speckle variance, as depicted in Figure 8. Structural data directly measures the depth of the sample points using time-delay characteristics, and the intensity of the backscattered light. Therefore, structural OCT captures the anatomical features of a tissue sample using the optical power density. The intensity reading of the photodetector after scanning the reference arm position, $I_{D}\left(z_{c,ij}\right)$, is [54]

(7) $$I_{D}\left(x_{i},y_{j},z_{c}\right)\propto S_{0}\sum_{c=1}^{C}\gamma (x_{i},y_{j},z_{c})\sqrt{R\left(x_{i},y_{j},z_{c}\right)R_{R}}cos(2kz_{c})$$

where $S_{0}$ is the spectrally integrated source power, $R\left(x_{i},y_{j},z_{c}\right)$ is the infrared reflectivity of the sample at depth $z_{c}$ for each location $\left(x_{i},y_{j}\right)$ within the raster scan and $R_{R}$ is the reflectivity of the reference mirror. γ is the coherence envelope at each spatial location in the raster scan, where γ is obtained from the inverse Fourier transform of the normalized power spectrum. k is termed the wavenumber, k=2π/λ, where λ is the wavelength of the emitted light. Meanwhile, variance OCT, specifically OCT-A, provides contrast to blood flow or bulk particle motion. This is possible due to the movement of the red blood cells that create signal variations within the individual OCT scans. Specifically, the variance measures how much the intensity of signal changes over time. One approach for this is the speckle variance is [64], given as

(8) $$I_{SV}\left(x_{i},y_{j},z_{c}\right)=\frac{1}{B}\sum_{b=1}^{B}{\left(I_{b}\left(x_{i},y_{j},z_{c}\right)-I_{mean}\left(x_{i},y_{j},z_{c}\right)\right)}^{2}$$

where B is the number of B-scans, $I_{b}\left(x_{i},y_{j},z_{c}\right)$ is the intensity at each indexing location of the bth scan and $I_{mean}\left(x_{i},y_{j},z_{c}\right)$ is the average intensity of the bth B-scan over the same set of pixels.

Utilizing structural OCT has many advantages, such as having high spatial resolution and faster acquisition times due to easy-to-process signals [65]. However, bulk particle motion can distort images by giving a grainy appearance [66]. One of the many benefits of OCT-A, or variance OCT, is that it provides excellent contrasting in vessels. It also does not require injection of dye for fluorescein angiography. However, it is more susceptible to motion artifacts, since bulk tissue motion can alter intensity variance. It also requires the triggering of multiple frames at the same location.

#### 2.3.3. Methods to Mitigate Artifacts and Better Analyze OCT-A Images

Correcting artifacts can be done through repeating OCT scans, but this can increase the SNR gap due to unpredictable patient motion [67]. Although, it should be noted that for the purpose of gathering skin VAD, this is simply not feasible due to the loading cycles induced onto the skin and the attempt to capture the swift reactive hyperemia response. Therefore, the methods to be introduced are for mitigation purposes and not remedial purposes.

##### Post-Processing Methods for Image Contrast Adjustment and Noise Reduction

Among post-processing strategies for artifact mitigation, contrast adjustment and noise reduction methods provide a foundational approach to improving image quality and reducing artifact interference. Motion-induced artifacts typically manifest as bright signal abnormalities that may be erroneously interpreted as patient vessels, confounding accurate vascular assessment and VAD calculation. Such motion artifacts can be seen in Figure 5 as thin, vertical, white lines throughout the image. Therefore, researchers have found that using adaptive thresholding to factor out the background signal enhanced the visibility of blood vessels that undergo bulk motion. This can reduce the signal produced from the blood flow, thus requiring contrast adjustments to enhance the blood flow detection [68]. Noise represents a fundamental challenge in OCT imaging, degrading image quality and potentially obscuring fine vascular details necessary for accurate diagnosis. Chen et al. [69] introduced a split-spectrum amplitude and phase-gradient angiography (SSAPGA) algorithm that employs the gradient of the phase difference and decorrelates the split-spectrum amplitude. Their results consisted of very small standard deviations over 15 experiments for vessel diameter index, blood vessel density, vessel connectivity, image contrast-to-noise ratio (CNR), and image signal-to-noise ratio (SNR), thus reducing image noise while maintaining the integrity of the VAD calculation. In another approach, Huang et al. [70] presented an SNR-adaptive time-series statistical characterization model of intensity and decorrelation. They found it to enhance the vessel visibility by identifying static voxels, labeled as surrounding tissue, using a spatio-temporal kernel size at a given SNR level and removing them from the image. Contrast-limited adaptive histogram equalization (CLAHE) offers another processing approach that enhances local image contrast while preventing the over-amplification of noise in relatively uniform regions [12].

##### Artificial Intelligence for OCT Image Adjustment

While conventional image adjustment techniques have proven useful, they require the use of an arbitrary user-selected threshold value. Artificial intelligence and machine learning approaches offer more adaptive methods for optimizing OCT image quality and reducing artifacts. Researchers can input images that they label satisfactory and train an artificial intelligence model to statistically identify characteristics to compare, adjust, and reconstruct test images to generate high-quality images. Given the computational expense of processing OCT-A image data, artificial neural networks have emerged as valuable tools for image extraction and artifact removal, significantly reducing reconstruction time compared to traditional methods. Liao et al. developed an IRU-Net neural network that improved the quality of reconstructed OCT-A images and reduced the signal processing speed by 83% [67]. Another approach for polarization-sensitive OCT (PS-OCT) reduces motion artifacts and surface reflectivity artifacts by training a network to label static tissue as noise and systematically remove it from the final image [71]. These advancements demonstrate the potential of artificial intelligence-based approaches to enhance OCT-A image reconstruction, reduce image artifacts, quickly reconstruct images, and enable more reliable analysis for clinical applications.

##### Optical Clearing Agents

While artificial intelligence methods address image quality through computational means, optical clearing agents offer a complementary approach by physically modifying tissue properties to enhance vessel visibility during image acquisition. Optical clearing agents represent a tissue preparation approach that enhances vessel visibility by temporarily reducing light scattering in biological tissues, improving imaging depth and contrast in OCT acquisitions. One such clearing agent suggested by [17] is a gel composed of fructose, polyethylene glycol, and thiazone (FPT) placed on the skin surface. It was found to allow for 0.16 mm of increased imaging depth. The FPT induces skin dehydration which reduces the light scattering coefficient by 40.5% and adjusts the refractive index mismatch by 25.3%, claiming a 5.83 × 10^−5^ cm/s optical clearing rate [19]. Optical clearing rate is the time it takes for a tissue to become fully transparent after the application of an optical clearing agent. Their findings demonstrate that optical clearing agents can significantly enhance OCT image quality during image acquisition, which effectively improves the ability to see the vascular structures within the skin.

##### Vessel Segmentation

**Figure 9:**
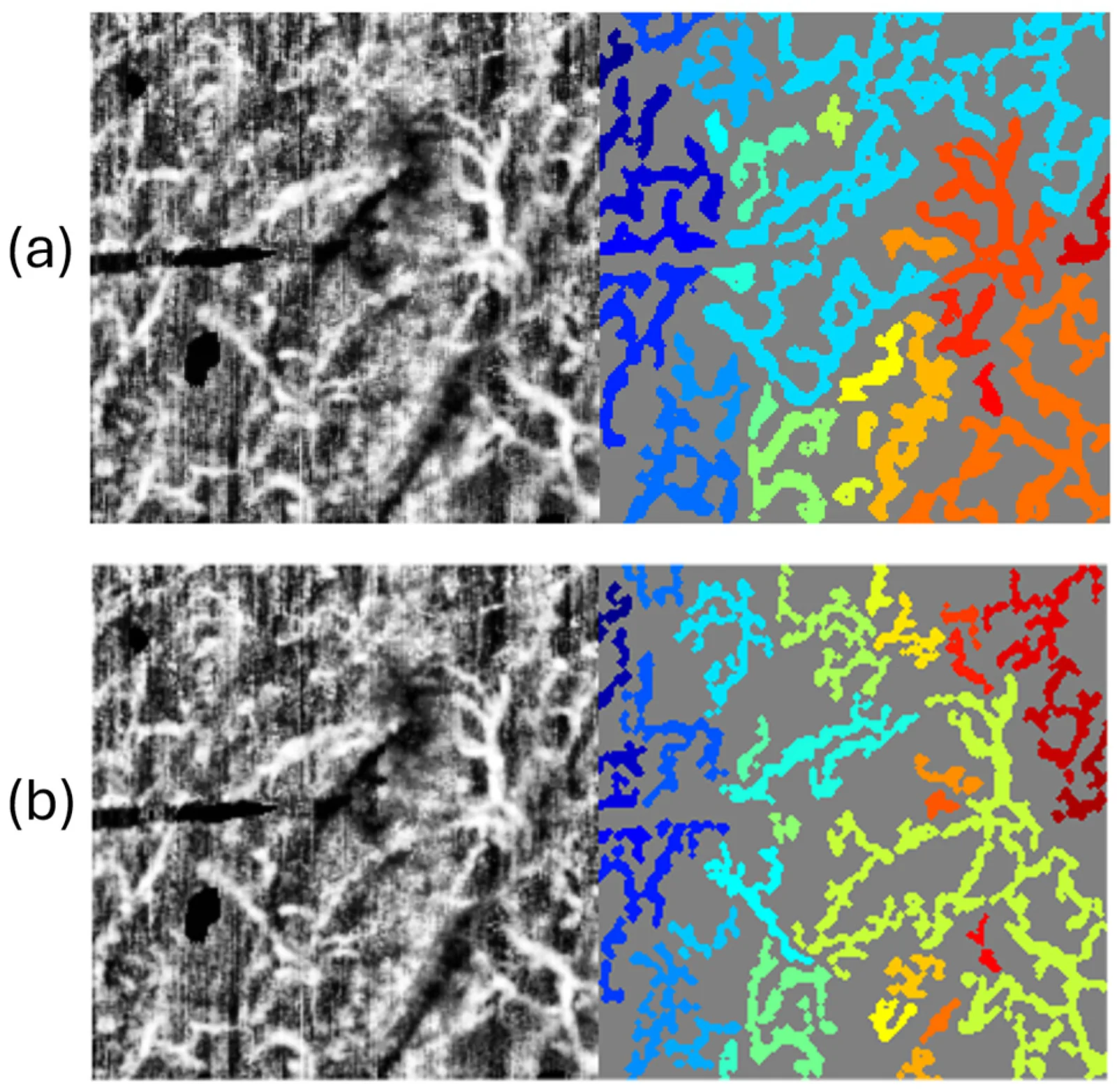
Vessel segmentation comparison: (**a**) poor segmentation extraction example and (**b**) proper vessel segmentation, where the color is only for distinguishing separate features.

Following noise reduction and artifact mitigation, vessel segmentation represents a critical step in identifying and delineating vascular structures from the processed OCT-A images. Vessel segmentation is the process of identifying and extracting vascular structures from OCT-A images. Accurate segmentation is essential for measuring VAD and other clinical skin metrics that are critical for diagnosing, and monitoring skin adaptation and vascular pathologies. The segmentation task is inherently challenging due to the complex branching morphology of dermal microvasculature and the presence of image noise and artifacts that can obscure true vascular structures. This can cause a misrepresentative VAD plot as shown in Figure 9. The misrepresentative segmentation image, Figure 9a, is after doubling the thresholding factor causing the algorithm to believe that a typical vessel diameter is 14 μm rather than the expected 7 μm. The more accurate segmentation, shown in Figure 9b, adjusted the thresholding factor to capture the expected vessel diameter of 7 μm.

**Figure 10:**
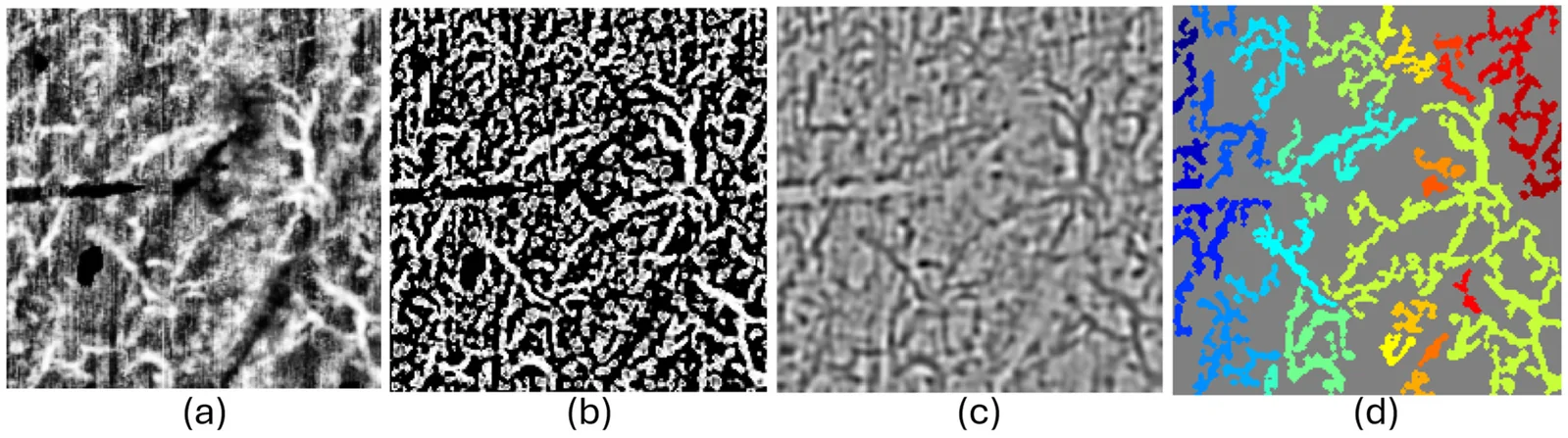
Vessel segmentation methods: (**a**) standard contrast adjustment, (**b**) Frangi Vesselness of the contrasted image, (**c**) Mexican Hat filter of the contrasted image, and (**d**) the extracted boundaries from a processed image.

The Frangi vesselness filter is an important algorithm that is based on the eigenvalues of the Hessian matrix and uses a function termed the vesselness function that normalizes the Hessian matrix, which is then convoluted with a Gaussian kernel [15]. A segmented image is then obtained by analysis of the vesselness function at different scales and summing the filter responses [15]. The Frangi vesselness filter is noted as a preprocessing step for vessel segmentation [14]. Using the Frangi vesselness filter, one obtains the results from the initial image Figure 10a to Figure 10b. Some authors have used an en face OCT-A image to highlight vascular patterns for clean data [72]. Another geometric approach is the Mexican hat filter which uses a Laplacian of Gaussian (LoG) operator that combines Gaussian smoothing with a second-order derivative for edge detection. The filter smoothens the edges and decreases background noise such as that shown in Figure 10c [13]. Another method for effective blood vessel segmentation is to use short-time principal component analysis (PCA) to decompose the image into low-rank matrices that represent slowly varying background skin tissue and into sparse matrices that represent blood vessels [73]. Combined with various preprocessing methods, vessel segmentation, as observed in Figure 10d, is a useful method for clear analysis of OCT-A images and allows for automated quantification of vessel characteristics such as VAD.

##### VAD Calculation and Estimation

**Figure 11:**
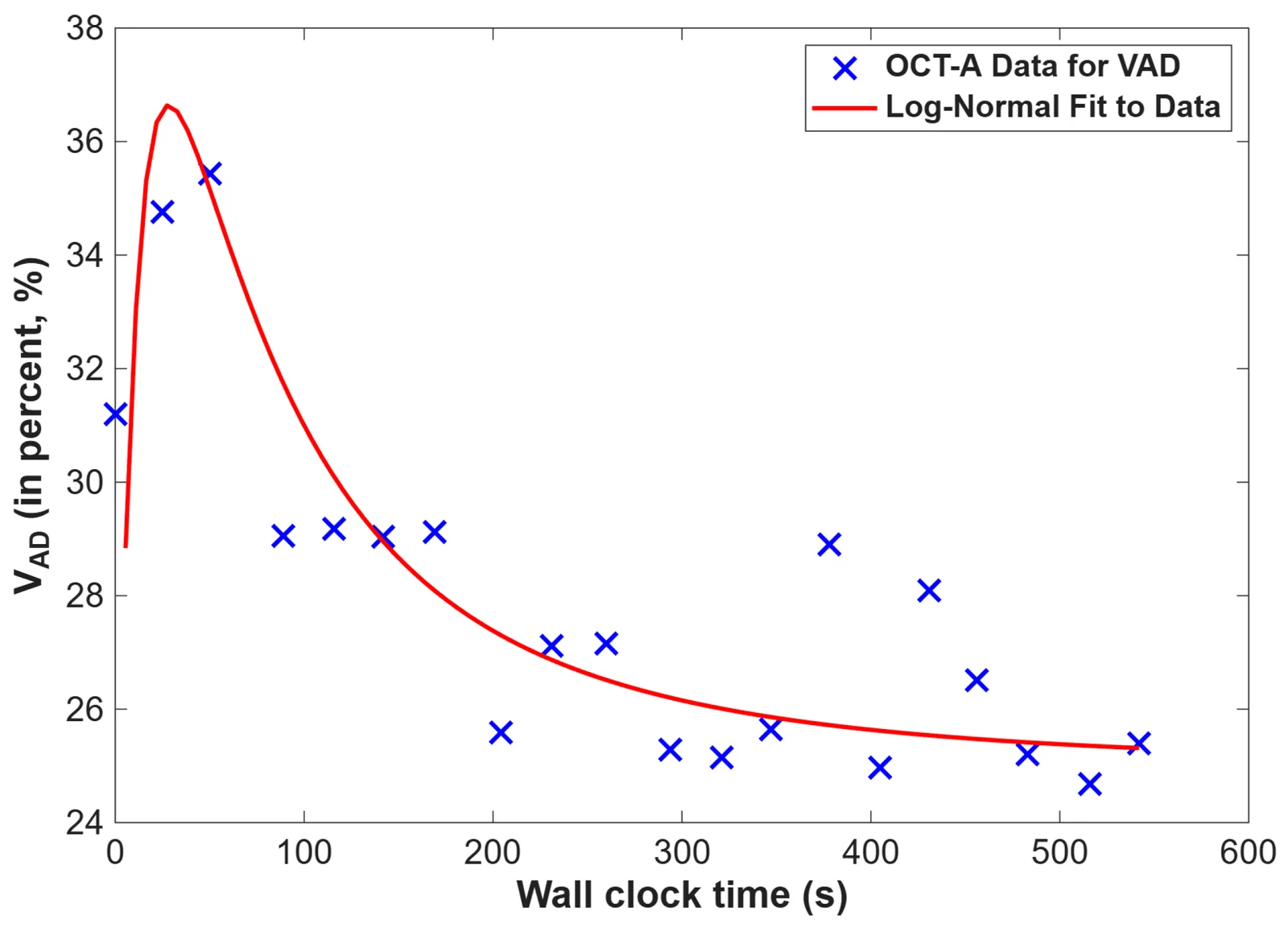
VAD plot with curve fit.

Using the post-processing methods listed above, a clear OCT-A image of the vessels is necessary due to the need to reliably segment and label the vessel geometries. After vessel segmentation, the total sum of pixels labeled as vessels is divided by the imaging window area minus objects blocking the view (i.e., hair shafts, scabbed wounds). The equation for calculating VAD is as follows:

$$VAD\left(t\right)=\frac{V_{A}\left(t\right)}{I_{A}-k_{A}(t)}\times 100\%$$

where $V_{A}$ is the sum of the pixels labeled as vessels, $I_{A}$ is the total imaging window area, and $k_{A}$ is the sum of the pixels labeled as throwaway objects. This VAD calculation occurs with every OCT-A image after dermal loading and is plotted over time, as shown in Figure 11, where a curve fit is applied to allow for VAD estimation at selected time periods.

## 3. Observations of the Benefits and Limitations of OCT-A and IR

OCT-A and IR imaging each possess their advantages and limitations when used for skin health assessment. Comparing OCT-A and IR to visual assessment based on observations found in the literature and in-clinic research offers important guidance for clinicians depending on their needs, budget, and medical personnel. Factors such as device cost, training requirements, diagnostic capability, and operator cost all influence the selection of an appropriate assessment tool. Table 2, shown below, summarizes our key findings from the reviewed literature by rating IR, OCT-A, and visual assessment on a basis of low, moderate, and high, as well as yes and no depending on the subject.

Some of the primary advantages of IR are its relatively moderate cost, minimal required operator training, and the rapid acquisition of data over a large field of view. By measuring skin surface temperature, IR can identify areas of localized skin tissue temperature profiles that may indicate developing skin damage. These characteristics make IR attractive for the possible use of routine screening and monitoring applications. However, IR tends to have a high sensitivity to environmental conditions such as humidity and ambient temperature fluctuations, but a relatively low sensitivity to patient movement.

In contrast, OCT-A provides more detailed information regarding tissue and vascular structures. Although OCT-A systems typically involve higher equipment costs and require greater expertise, they can offer access to a wider range of skin adaptation and skin breakdown biomarkers. In addition to visualizing vascular structure and their areal changes to transient conditions such as blood perfusion, OCT-A can provide information such as epidermal thickness, tissue morphology, and collagen structure. These biomarkers can allow for accurate, faster, and more detailed early detection of skin breakdown and skin adaptation as compared to IR and visual assessment.

Visual assessment remains the most common and cheapest method in clinical practice. Experienced clinicians notice signs of skin breakdown such as inflammation, ulceration, and blistering. However, these indicators tend to appear only after significant skin damage occurs, and the corrective action invariably requires the patient to decrease their prosthesis usage.

Collectively, these three methods have their own advantages and drawbacks, as detailed in Table 2. In short, visual assessment provides a fast and low-cost method of monitoring and detecting skin breakdown but does not provide early pre-symptomatic detection. Conversely, OCT-A offers highly detailed information on structural and vascular changes that reveal early signs of skin breakdown and adaptation but can drive the cost levels up. The spatial region of interest is also quite low compared to IR and may require multiple tests to identify regions of concern. Lastly, IR offers quantitative results on skin adaptation and breakdown indicators unlike visual assessment. When compared to OCT-A, IR has less information presented to the clinician, although the costs are typically lower.

## 4. Future Work

Improvements in artifact-removal algorithms, processing speed, and image acquisition protocols would not only enhance standalone OCT performance but also provide higher-quality training data and inputs for artificial intelligence models. Faster image reconstruction and automatic artifact correction would enable immediate clinical decision-making while artificial intelligence-assisted diagnostics could simultaneously identify subtle vascular changes indicative of early skin breakdown that might escape human observation. IR cameras are subject to more physical improvements rather than software improvements. However, comparing IR and OCT on the same specimen to see how closely they relate to each other could establish more trustworthy results for a clinician. Furthermore, the automation of thresholding factors based on physics principles on the various filtration methods before segmentation would greatly reduce the time for the clinician during the post-processing phase and increase the repeatability of vessel segmentation. The integration of these technological advancements could fundamentally transform prosthetic care from reactive treatment of skin breakdown to proactive prevention, ultimately improving outcomes and quality of life for patients.

As artificial intelligence becomes more prominent across all industries, the usage of this tool can be promising for clinical diagnosis assistance in skin breakdown detection and feature labeling. Currently, artificial intelligence is heavily researched within ophthalmology, which commonly utilizes OCT. Pathologies such as macular degeneration can be accurately identified by deep-learning models and can even assess different stages of macular degeneration [74,75]. While artificial intelligence shows tremendous promise for providing macular diagnostic assistance, its effectiveness in skin breakdown diagnosis depends critically on continued advancements in the underlying OCT technology itself.

## 5. Conclusions

Despite the critical importance of early skin breakdown detection, clinicians currently have a limited array of tools available for objectively assessing skin in relation to prosthetic interfaces. Clinicians currently rely on visual assessment, patient input, and palpation to assess indicators of skin breakdown. Providing tools that output quantitative measurements early in skin breakdown events can improve patient outcomes, quality of life, and healthcare costs. Thermal imaging and OCT offer valuable information, but both have their limitations for use in a clinical setting. Thermal imaging cannot output subcutaneous geometrical features such as microvessels, epidermal thickness, and collagen content, which can indicate information to the clinician to diagnose pressure injury on the skin. However, these are features that OCT can provide for the clinician. Thermal imaging only provides surface-level information and can face false readings given ambient temperature fluctuations, relative humidity fluctuations, and temperature profile non-uniformity. These issues can be improved by providing non-uniformity correction methods and managing ambient conditions within the environment and IR software. However, the time-to-peak temperature profile tends to be more repeatable than the vascular arial density from OCT-A, offering a uniform time-to-peak response across different scans, which can provide a valuable indicator for skin breakdown assessment. OCT-A provides the clinician with vessel dynamics and can be used to assess other skin conditions. Using vessel area density (VAD), OCT-A can present the clinician with measurable results that can be used to assess early skin breakdown. However, OCT-A faces artifacts caused by patient movement, optical phenomena, and incorrect test setups. Therefore, software methods, artificial intelligence, patient movement management, and optical clearing agents can aid in reducing imaging artifacts, allowing for reliable vessel segmentation and VAD calculation. The best noninvasive method for identifying skin breakdown which balances information gained and predictive capability with training required to operate and cost remains unknown. As highlighted in the present manuscript, future work should focus on direct comparisons of techniques to better evaluate their respective advantages and disadvantages, thus enabling these advanced methods for clinical usage. Although multiple algorithms are being proposed for converting the captured data from OCT and IR, there is a lack of standardized data sets allowing comparisons between methods, which prevents the validation of each methodology. To aid in the diagnosis of skin breakdown, there is the potential for the use of artificial intelligence-assisted analysis such as currently being pursued in the use of macular degeneration detection through OCT.

## 6. Disclosures and Disclaimers

There are no conflicts of interest to be disclosed. The views, opinions, and/or findings expressed in this manuscript are those of the authors and do not necessarily reflect the views of the Henry M. Jackson Foundation for the Advancement of Military Medicine Inc., Defense Health Agency, Department of War, or any other U.S. government agency, and should not be construed as an official DoW/Army position, policy or decision unless so designated by other documentation. No official endorsement should be made. Reference herein to any specific commercial products, process, or service by trade name, trademark, manufacturer, or otherwise does not necessarily constitute or imply its endorsement, recommendation, or favoring by the U.S. Government.

Some of the authors are employees of the U.S. Government. This work was prepared as part of their official duties. Title 17, U.S.C., Section 105 provides that copyright protection under this title is not available for any work of the U.S. Government. Title 17, U.S.C., Section 101 defines a U.S. Government work as a work prepared by an employee of the U.S. Government as part of that person’s official duties.

## Figures and Tables

**Table 1:** Common OCT-A artifacts and their definitions.

Artifact	Definition
Decentration	When the optical center of the lens is not aligned with the object of interest, causing an inaccurate representation of the object [58,59].
Defocus	When the definition of the OCT image is reduced due to focusing issues [59].
Shadow	When an obstacle that blocks the propagation of light impedes the visualization of details within the region of interest [59,60].
Z-offset	When OCT scans are vertically displaced due to faulty head placement [59,61].
Mirror	During Fourier-domain detection, OCT images are symmetrical around the zero-time-delay line because the Fourier domain cannot distinguish positive from negative time delays [62].
Out of Register	When the scan is shifted superiorly or inferiorly, some of the layers of depth are not fully imaged [63].
Cut Edge	When the edge of the scan is cut off due to an incomplete scan.
Degraded	When the image lacks detail due to poor image acquisition [63].

**Table 2:** Comparison of visual assessment, IR, and OCT-A.

Subject	Visual Assessment	IR	OCT-A
Operator Cost	Moderate	High	High
Operator Training	Moderate	Moderate	High
Infrastructure Cost	Low	Moderate	High
Early Detection of Tissue Degradation	No	Yes [6]	Yes [8]
Ability to Identify Individual Vascular Regions	No	No	Yes
Requires Post-Processing for Image Analysis	No	Yes	Yes
Sensitivity to Environmental Surroundings	Low	High	Moderate
Size of Region of Inspection	Large	Large	Small
Sensitivity to Vascular Flow Rate	Low	Moderate	High
Ability to Push New Analysis Techniques	No	Yes	Yes
Identify Epidermal Thickness	No	No	Yes [27]
Ability to Detect Collagen Levels	No	No	Yes [44]

## Data Availability

The raw data supporting the conclusions of this article will be made available by the authors on request.
